# Comparing Patient Simulation With a Humanoid Robot or a Human Actor in Terms of Training Success and Acceptance: Pilot Questionnaire Study

**DOI:** 10.2196/70363

**Published:** 2025-12-05

**Authors:** Patricia Schwarz, Sandra Hellmers, Sebastian Spanknebel, Diana Immel, Rene Hurlemann, Andreas Hein

**Affiliations:** 1 Assistance Systems and Medical Device Technology Department of Health Services Research Carl von Ossietzky Universität Oldenburg Oldenburg Germany; 2 School of Medicine and Health Sciences Department of Psychiatry and Psychotherapy Carl von Ossietzky Universität Oldenburg Oldenburg Germany

**Keywords:** humanoid robot, simulated patient, robot patient, medical education, psychiatry, schizophrenia, artificial intelligence, AI, psychiatric nursing

## Abstract

**Background:**

The addition of simulated patients to medical and nursing training makes it possible to create a link between theory and practice. This makes what has been learned more realistic and allows the complexity and multilayered nature of many illnesses to be reflected in a real-life setting. However, the selection, training, and supervision of actors as simulated patients is time consuming and expensive. In this study, we investigated how differently students and nurses perceive 2 different methods of patient simulation.

**Objective:**

The aim of this pilot study was to investigate whether patient behavior simulated by a humanoid robot is comparable to patient simulation by actors in videos in terms of training success and user acceptance. Participants were asked to recognize the symptoms presented by the humanoid robot and make a diagnosis. For comparison purposes, we asked a second group of participants to make a diagnosis based on a video featuring a human patient actor.

**Methods:**

We asked the participants (medical students and nursing staff; N=21) to conduct a psychopathological assessment. Group 1 (n=11) used the humanoid robot as a patient simulator, and group 2 (n=10) watched the identical symptoms in a video with a human actor as patient.

**Results:**

The participants had a mean age of 28.7 (SD 3.5) years. The students were in their sixth semester and had, on average, 7.6 (SD 3.3) years of professional experience in the medical field. The correct diagnosis was made 90% (9/10) of the time based on the video with the human patient actor and 91% (10/11) of the time based on the robot. One participant in each group made the wrong diagnosis, constituting a total error rate of 10% (2/21). In general, participants with the humanoid robot as patient simulator felt more confident that their diagnosis was correct compared to those with the human actor as patient (humanoid robot: 9/11, 82% were neutral to very confident and 2/11, 18% were uncertain to very uncertain; human actor: 9/10, 90% were neutral to very confident and 1/10, 10% were uncertain or unsure).

**Conclusions:**

The simulations of the human actor in the video were judged to be more realistic overall than those of the humanoid robot as patient simulator. However, the differences between the simulation methods in relation to the result (diagnosis) were very small. The results of our pilot study show a good performance of the robot in the simulation of selected psychiatric patient cases. We conclude that a humanoid robot could be a useful addition to patient simulators in medical education and discuss future directions.

## Introduction

### Background

Learning and practicing the collection of psychopathological findings is an essential aspect of teaching as part of medical studies. This practice summarizes the results of a psychiatric examination and forms the basis for diagnostic decisions and therapeutic measures [1]. Assessing findings is an important part of physicians’ everyday work as they spend up to 80% of their time communicating with patients [2]. In the field of psychiatry in general, having various training opportunities is important so that students can understand complex behaviors and use medical conversation techniques. The ability to determine a diagnosis can be learned in practical training units. In addition to theoretical lessons, human patient actors are used to simulate realistic situations [3]. The role of the patient is played by human actors who have been prepared for the role in advance by the lecturer and a corresponding script. Symptoms can be objectively visible or subjectively experienced [4]. Recognition of symptoms and correct classification requires knowledge of psychopathological terminology and training in perception [5]. Some signs are almost pathognomonic for a diagnosis, but most signs can also occur with various clinical symptoms and sometimes in healthy people [6].

Recognizing and identifying symptoms is crucial not only for medical students but also for nurses. Accurate symptom assessment forms the basis for effective interdisciplinary collaboration. Nurses are often the first to notice changes in a patient’s health status and, thus, provide crucial information for medical decision-making. In multidisciplinary teams, such as in psychiatry or intensive care, nurses contribute to the early detection of complications and the adjustment of treatment strategies through their continuous observation of patients. Trained diagnostic perception among nursing staff not only improves the quality of care but also strengthens communication between professional groups and promotes joint, patient-centered action.

### Previous Training Methods and Their Limitations

Human actors have traditionally simulated patient behavior by following scripts, but their performance can still vary due to environmental factors, time, conditions, or participant interactions [7]. The representation of difficult and complex patient behavior by human actors is often stressful and difficult to standardize, leading to improvised and, therefore, varying interactions between actor and student. In addition, the preparation of roles and their variations is very time-consuming [8]. These seminars also require a great organizational and financial effort, which allows students only very limited practice opportunities [2]. Role-playing with fellow students is an alternative to acting patients in videos but has drawbacks such as familiarity, lack of feedback training, and limited empathy. In particular, complex psychiatric behaviors are difficult to simulate realistically using case studies or videos [9].

### Potential of Humanoid Robots as Simulated Patients

So-called patient simulators [10] have revolutionized medical training, assessment, and research for several reasons. They can be used to recreate high-risk situations in controlled and safe environments. Patient simulators are consistent across users because their performance is not affected by individual trainee differences or other environmental variables that influence human behavior. This is especially true given recent advances in robotics [11]. New-generation humanoid robots attract interest due to their humanlike appearance and social-interactive capabilities, enabling physical interaction as inanimate patient simulators [12,13]. The abilities and strengths of modern humanoid robots are particularly well suited to the representation of facial expressions and gestures. Therefore, when designing the training scenario, we focused on the imitation of psychiatric disorders by the robot. In a prestudy, we investigated the technical requirements and acceptance of the students [14].

In the current simulated patient program at the University of Oldenburg, the clinical picture of schizophrenia is also practiced with acting patients. Existing records of acting patients enabled the robot to reconstruct patient behavior. In collaboration with colleagues from the Department of Psychiatry, the clinical picture of schizophrenia was selected as a clinical case study for the robot simulated patient in our training scenario. In 2021, approximately 0.3% of the population worldwide had schizophrenia; this value has been stable in recent decades [15]. Commonly affected symptom domains include thought processes, emotions, body perception, and interpersonal contact, often leading to difficulties distinguishing reality from subjective experience [16,17]. Schizophrenia can manifest in a wide variety of symptoms. We will use some example symptoms to examine the feasibility of patient simulation by humanoid robots.

### Aim of the Study

On the basis of our previous studies [18,19] and the limitations of current humanoid robots for the patient simulation program, we developed our own approach.

The aim of this pilot study was to investigate whether patient behavior simulated by a humanoid robot is comparable to patient simulation by actors in videos in terms of training success and user acceptance. Two groups of participants with a medical background were asked to conduct a psychopathological assessment on the humanoid robot (group 1-R) and the human actor in the video (group 2-V) and make a diagnosis based on this. The participants were instructed to recognize the symptoms presented by the humanoid robot or the human actor and make a diagnosis.

The training concept is generally based on the taxonomy by Bloom [20] and promotes a cognitive learning process. On this basis, a study design was developed that measured the diagnostic performance and subjective perception of participants when using a humanoid robot compared to a video with an actor playing a patient. To evaluate training outcomes, this pilot study examined participants’ acceptance of the robot as a learning tool and their perception of the robot’s simulated behavior realism. The following research questions were answered:

1. Symptom identification: are the participants in both groups able to identify the symptoms presented by the given simulated patient?
2. Correct and incorrect diagnoses and influencing factors: what is the percentage of correct and incorrect diagnoses in the 2 groups?
3. Reliability of diagnosis: do the participants consider their own diagnoses to be reliable (self-assessment), and were there differences in relation to each group?
4. Realism in patient presentation: is it possible to simulate and present the patient behavior shown by the humanoid robot as realistically as possible?

The first research question investigates whether the participants in both groups can recognize the symptoms presented by the simulated patient, whether there is a difference in the ability to make a correct diagnosis between groups 1-R and 2-V, and how realistic the presented symptoms were from the participants’ perspective. The second research question determines the proportion of correct and incorrect diagnoses in both groups, as well as criteria for determining user acceptance of the robot-assisted procedure. The comparison between robot and actor patients enabled an objective assessment of standardization, reproducibility, and acceptance. The third research question examines whether participants consider their own diagnoses to be reliable and whether this self-assessment differs between the 2 groups. The fourth research question observes whether a humanoid robot can realistically represent patient behavior. Technical limitations such as limited facial expressions and mouth movements were taken into account, and the transferability of complex behaviors, such as those associated with schizophrenia, to the robot was evaluated.

## Methods

### Overview

The Study Design section describes the study procedure. As part of the pilot study, the robot presented a use case through facial expressions, gestures, and speech. The Case Design section describes the use case that we evaluated. For this purpose, the most important requirements for the humanoid robot as a simulated patient were analyzed (for a full analysis of requirements, refer to Multimedia Appendix 1).

### Study Design

**Figure 1:**
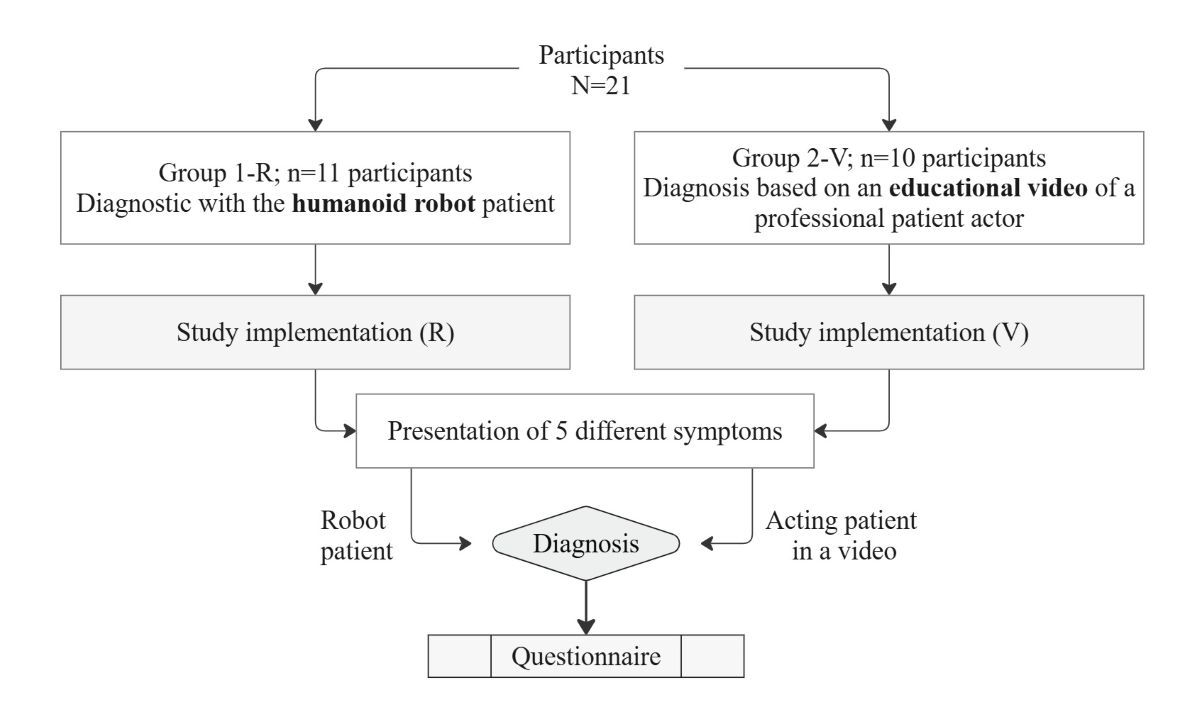
Flowchart of the study procedure and individual steps. R: robot; V: video.

One objective was to investigate whether participants from the medical field are able to identify psychopathological symptoms in a simulated patient. For this purpose, both a humanoid robot as a simulated patient and videos of an actor patient were used, and 2 groups of participants were formed: group 1-R conducted the assessment using the humanoid robot patient, and group 2-V did so using the human patient actor in the video. The participants were assigned to 1 of the 2 groups through block randomization (Figure 1).

At the beginning of the pilot study, participant inclusion and exclusion criteria were applied. Students or professionals in medicine, nursing, or psychology aged ≥18 years were eligible to participate; individuals who did not understand the study content or were unable to perform the exercises due to severe visual impairments were excluded. Participants were then informed about the aim and procedure of the study and gave their consent.

The procedure was explained to the participants, and the aim of the study was presented. Following the introduction, the participants were asked to complete a questionnaire (Multimedia Appendix 2). This involved a standardized assessment and documentation of psychopathological findings (for the psychopathological categories and associated symptoms covered in this study, see Multimedia Appendix 3). Depending on group allocation, participants observed selected symptom presentations in the robot or the human actor. They conducted a psychopathological assessment after up to 5 symptoms, which were identical in content and sequence for both simulations, each lasting 0.3 to 2 minutes. Participants could diagnose early if they were confident and take notes during the presentations. Following the simulations, participants recorded their findings and completed a questionnaire. Figure 1 illustrates the study process and steps.

### Case Design

#### Overview

**Figure 2:**
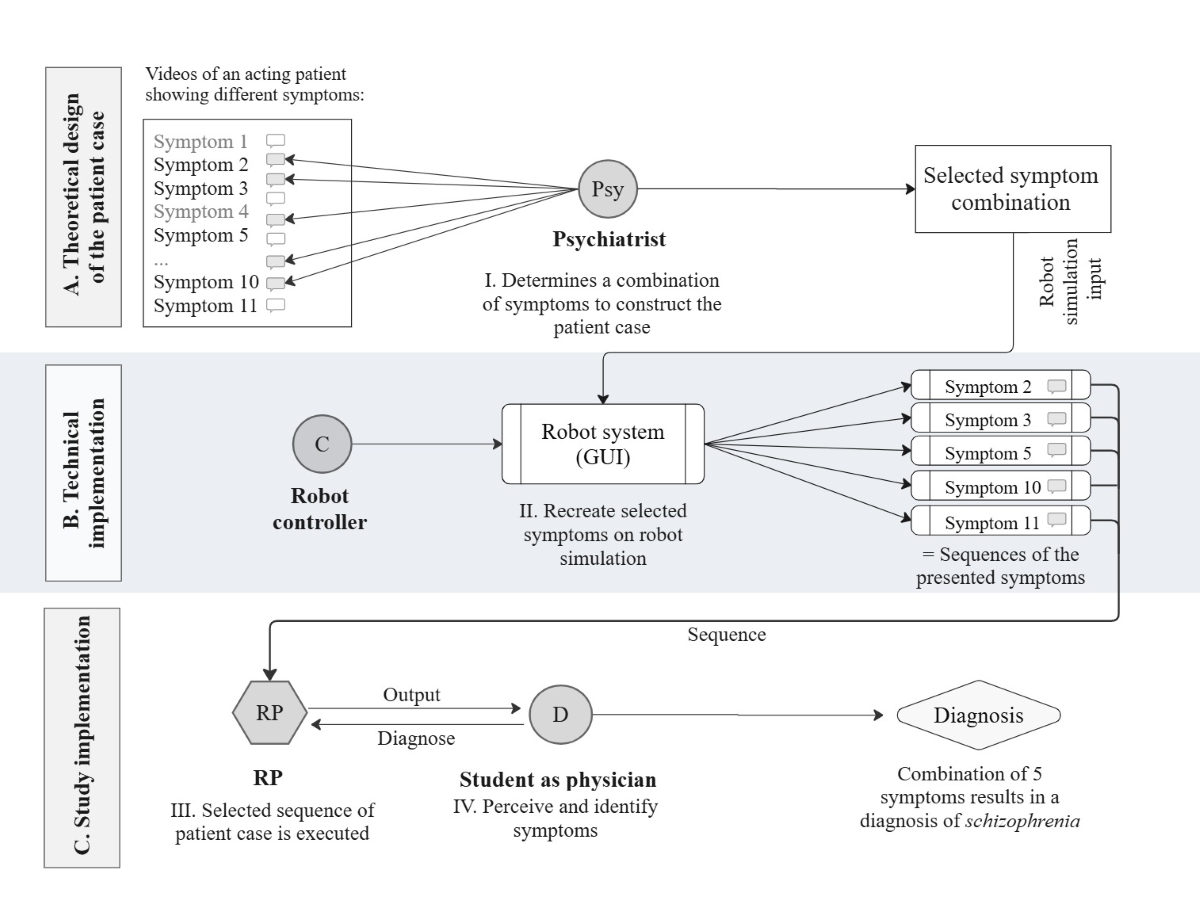
Overview of the components required to conduct a psychopathological assessment using a humanoid robot patient (RP). GUI: graphical user interface.

The overview graphic in Figure 2 shows the procedure and the individual components required to conduct a psychopathological assessment using a humanoid robot patient. The three phases were (1) theoretical design of the patient case, (2) technical implementation, and (3) study implementation.

Videos of actor patients presenting various psychopathological symptoms served as a reproducible and precise basis for the robot simulation. This approach enabled direct comparison between the robot and video simulations by participants. In total, 12 videos depicting different symptom combinations, creating distinct clinical profiles, were used to develop the humanoid robot patient. This provided a very detailed basis for transferring the behavior shown in the video to the robot step by step. The individual symptoms were selected by an expert (psychiatrist) and represented the diagnosis of schizophrenia.

All data were transferred to a simulation via a graphical user interface (Figure 2). This was done by reproducing the actors’ mood, thoughts, and feelings (as an audio track). In combination with matching facial expressions and gestures (as movement sequences), this resulted in a sequence of symptom representations. The outcome was 5 different sequences, each mapping the content of the videos (the actors’ presentation of symptoms) onto the robot. Altogether, these elements produced the robotic presentation of the patient case (Figure 2).

In the next step, the specific audio and movements for each symptom were transferred to the robot management system. Movements, speech, and facial expressions from the video (section I in Figure 2) were transferred the robot’s graphical user interface (section II in Figure 2) and then played back on the humanoid robot (section III in Figure 2). Thus, the videos served as *input* for the programmed sequence, and the simulation result was the *output* of the robot.

The individual symptoms were stored in the robot management system and, thus, could be presented in various combinations by the humanoid robot. The robot exhibited the programmed movements and spoke to the participant acting as the patient. The participant (in the role of the physician) observed the robot patient, listened to the symptom descriptions, and then attempted to make the appropriate diagnosis based on the presented symptoms. For each symptom of schizophrenia—flight of ideas, withdrawal of thoughts, delusions, delusional mood, and thought insertion—there was a short video showing a typical scene with a human actor portraying the patient.

#### Materials

**Figure 3:**
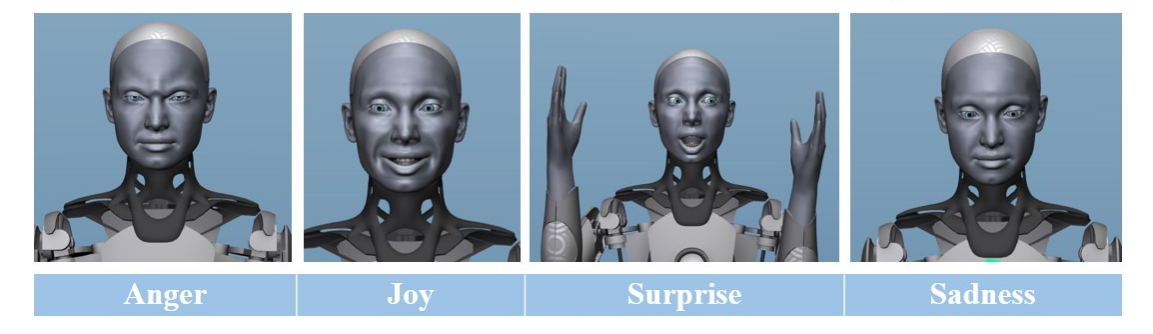
Ameca showing different emotions: anger, joy, surprise, and sadness.

The humanoid robot (Ameca; Engineered Arts) that we used in our study was specially designed as a platform for the development of future robot technologies and, therefore, is very well suited as a humanoid robot platform for human-robot interaction [20]. Facial expressions, movements, and the style of speech of patients can be programmed in the robot and, thus, imitated. Ameca can show different emotions and gestures, such as anger, joy, surprise, or sadness (Figure 3).

#### Participants

The pilot study included both medical and nursing students to capture diverse perspectives on patient simulation effectiveness. These groups differ in education, clinical experience, and professional roles, which may affect their symptom perception and diagnostic approaches. Including both cohorts enhanced the generalizability of the results across health care professions and allowed for analysis of how educational background influences symptom identification. Evaluating simulation performance across these groups helped identify specific strengths and limitations, guiding targeted improvements in patient simulation.

Flyers and the student portal were used to recruit students of medicine, psychology, or nursing. General information about the study and contact details of the study director were published on the student portal. Interested individuals had the opportunity to voluntarily register as study participants by contacting the study director via email. Participation was open to students aged >18 years who met the inclusion criteria.

A total of 21 participants were recruited. All participants gave consent for their data to be used in the study. The sessions took place in a university laboratory equipped with necessary assessment materials, including a table, chairs, humanoid robot, screen, psychopathology note sheets, and questionnaires. Participants were randomly assigned to 1 of 2 groups.

#### Questionnaire

The self-developed questionnaire contained 5 questions (for the full questionnaire, refer to Multimedia Appendix 2 in accordance with research question 1, question 2 of the questionnaire asked whether all symptoms could be identified and how many symptoms the participants viewed before making their diagnosis (question 3). To address research question 2, the diagnosis made was recorded (question 5). Subsequently, the diagnosis made was checked by experts and categorized as correct or incorrect. The questionnaire also recorded the age of the participants, their occupational group (medical student or nurse), and their professional experience in the medical field. In this way, the effect of these data on the correct or incorrect diagnosis was investigated. Question 4 measured how certain the participants were that the diagnosis they had given was correct. This answered research question 3. To answer research question 4, the results of questions 1 and 2 from the questionnaire, as well as the expert review, were used.

### Ethical Considerations

The Commission for Research Impact Assessment and Ethics at Carl von Ossietzky University of Oldenburg reviewed and approved this study (2024-016). The participants were fully informed about the study objectives and methodology. General participant information about the study was provided to the participants well in advance of the study start date. After the participants had been informed, their consent was obtained. The consent form contained all necessary information and was attached to the ethics application. The participants were informed about the procedures and objective of the study via a participant information sheet (sent via email). They had at least 1 day to decide to participate. Participants did not experience any consequences if they withdrew. There was no financial compensation for participating in the study. The privacy and confidentiality of the participants’ data generated or analyzed during the study will be protected.

### Data Collection and Analyses

The data were collected and processed anonymously using a code. The code list will be destroyed once data analysis has been completed. The data collected as part of this study will be used exclusively for research purposes.

In this study, descriptive data were collected using a questionnaire to evaluate the training method. The questionnaire contained both closed-ended questions (multiple-choice or dichotomous answer formats) and open-ended questions that gave participants the opportunity to formulate their answers freely. The aim of this survey method was to gain an overview of the existing attitudes, experiences, and perceptions of the respondents. The data were evaluated using the statistical software JASP (The JASP Team).

## Results

### Characteristics of the Participants

In total, 21 participants (n=4, 19% male and n=17, 81% female) took part in this study. All of them had a medical background (n=10, 48% medical students and n=11, 52% nurses; 15 (71%) participants stated that they had medical experience in practice, and 6 (29%) participants stated that they had no experience in practice. Table 1 shows the professional background, age, sex, work experience, and given diagnoses of the participants.

**Table 1:** Characteristics of the participants (N=21).

Characteristic			Values
Age (y), mean (SD)			28.7 (3.5)
**Sex, n (%)**			
	Male	4 (19)	
	Female	17 (81)	
**Profession, n (%)**			
	Medical student	10 (48)	
	Nurse	11 (52)	
Professional experience (y), mean (SD)			7.6 (3.3)
**Professional medical experience in practice, n (%)^a^**			
	Yes	15 (71)	
	No	6 (29)	
**Diagnosis rating, n (%)**			
	Correct	19 (90)	
	Incorrect	2 (10)	
**Given diagnosis, n (%)**			
	Schizophrenia or paranoid schizophrenia	17 (81)	
	Psychosis	2 (10)	
	Anxiety disorder	1 (5)	
	Delusions	1 (5)	

^a^Does the participant have a professional background in the medical/nursing field?

In agreement with experts in the field of psychiatry, the diagnoses of schizophrenia, paranoid schizophrenia, and psychosis were determined to be correct. The diagnoses of anxiety disorder and delusion were rated as incorrect.

### Survey Results

Table 2 summarizes the responses to the questionnaire. Most participants rated the human actor patient video as more realistic, with 80% (8/10) selecting “very realistic” and 20% (2/10) selecting “realistic.” In contrast, ratings for the robot patient were more varied: 27% (3/11) rated it as “very realistic,” 45% (5/11) rated it as “realistic,” and 27% (3/11) rated it as “neutral.”

**Table 2:** Survey results by group.

		Humanoid robot simulation (group 1; n=11)	Video simulation (group 2; n=10)
**Realism of patient simulation^a^**			
	1=very realistic, n (%)	3 (27)	8 (80)
	2=realistic, n (%)	5 (45)	2 (20)
	3=neutral, n (%)	3 (27)	0 (0)
	4=unrealistic, n (%)	0 (0)	0 (0)
	5=very unrealistic, n (%)	0 (0)	0 (0)
	Score, mean (SD)	2 (0.74)	1.2 (0.4)
**All symptoms identified^b^, n (%)**			
	Yes	10 (91)	9 (90)
	No	1 (9)	1 (10)
**Number of symptoms presented until diagnosis was made^c^**			
	2, n (%)	2 (18)	2 (20)
	3, n (%)	5 (45)	1 (10)
	5, n (%)	4 (36)	7 (70)
	Mean (SD)	3.5 (1.16)	4.2 (1.3)
**Self-confidence in diagnosis^d^**			
	1=very sure, n (%)	8 (73)	4 (40)
	2=sure, n (%)	1 (9)	5 (50)
	3=neutral, n (%)	0 (0)	1 (10)
	4=unsure, n (%)	1 (9)	1 (10)
	5=very unsure, n (%)	1 (9)	0 (0)
	Score, mean (SD)	1.73 (1.35)	1.9 (0.94)
Correct diagnosis, n (%)		10 (91)	9 (90)
Incorrect diagnosis, n (%)		1 (9)	1 (10)

^a^“How realistically were the symptoms presented?”

^b^“Were you able to identify all the symptoms presented?”

^c^“How many symptoms were presented to you by the simulated patient before you made a diagnosis?”

^d^“How sure are you that your diagnosis is correct?”

During the patient simulation by the robot, 18% (2/11) of the participants made the diagnosis after only 2 presented symptoms, and 45% (5/11) of the participants made the diagnosis after 3 presented symptoms. The other participants had all 5 symptoms presented to them before they made a diagnosis (Table 2). In the patient simulation by the human actor in the video, 20% (2/10) of the participants made a diagnosis after 2 symptoms were presented, and 10% (1/10) of the participants made the diagnosis after 3 symptoms. The remaining 70% (7/10) of the participants were shown all 5 symptoms before they made a diagnosis. In total, 90% (9/10) of the participants were able to identify all symptoms shown in the video. A total of 91% (10/11) of the participants were able to identify all symptoms shown by the robot.

The participants who diagnosed the actor patient reported that they were neutral (1/10, 10%), sure (4/10, 40%), or very sure (4/10, 40%) of the correct diagnosis. The participants who diagnosed the robot patient said that they were very sure (8/11, 73%), sure (1/11, 9%), unsure (1/11, 9%), or very unsure (1/11, 9%). Finally, we also evaluated the diagnoses made by the participants and checked whether these were correct. Overall, 90% (19/21) of the participants made the correct diagnosis, and 10% (2/21) made an incorrect diagnosis.

### Comparison Between the Established Procedure and the New Procedure

**Figure 4:**
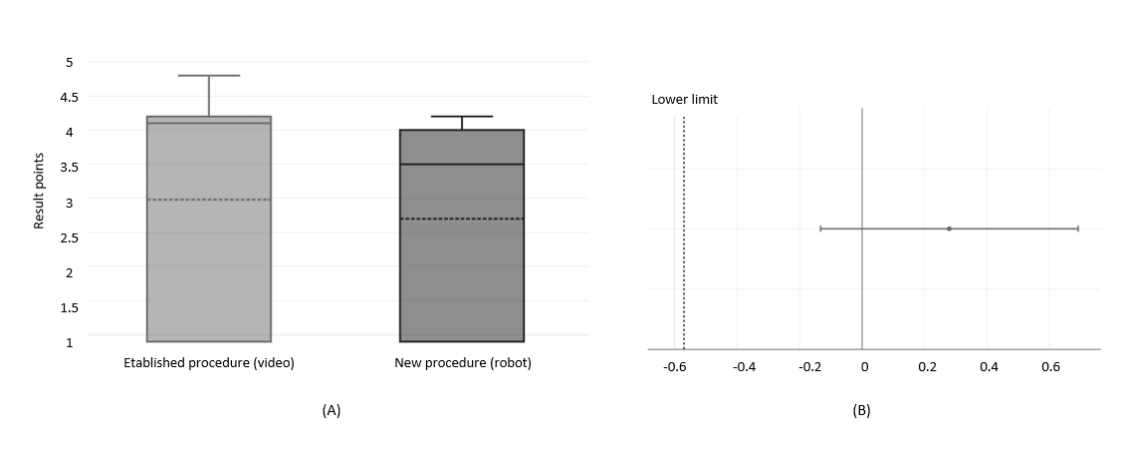
Feasibility and usability study: comparison between the results of the established procedure and the new procedure (A) and CIs (B).

Another aspect that was examined was whether the new procedure with the humanoid robot patient was worse or better than using the video with the actor patient (standard procedure). For this purpose, a limit must be defined in advance. In our case, we set a maximum deviation of 20% from the standard procedure. We calculated the deviation based on the average values of the questionnaire responses (Figure 4; average values: Multimedia Appendix 3).

## Discussion

### Principal Findings

The results show that, although participants found the video simulation more realistic, diagnostic accuracy and self-confidence were comparably high in both groups. Thus, the humanoid robot was able to successfully represent patient behavior in a way that enabled reliable symptom assessment and diagnosis, making it appear to be a suitable learning tool for patient simulation.

### Interpretations

The responses from participants in group 1 (robot patient) were more variable. Human actors can portray patients more realistically because they can authentically use complex emotions and subtle nonverbal signals, such as facial expressions, gestures, and body language. These subtle forms of expression are difficult for robots to implement due to technical limitations such as movement speed and flexibility. Humans also adapt their portrayal to reactions and context depending on the situation, which is currently only possible to a limited extent in robots.

The results indicate that only 1 participant per group made an incorrect diagnosis, showing minimal difference between groups. Although human actors’ realistic symptom presentation, nonverbal cues, interactive flexibility, and emotional credibility may aid diagnosis accuracy, overall results for the robot and video simulations were comparable. This suggests that the robot’s patient portrayal was not significantly inferior to the human actor videos. Using a 20% deviation criterion, the robot simulation was deemed noninferior to the standard procedure, demonstrating equivalence in realism, symptom recognition, and diagnostic accuracy. Thus, in our pilot study, the training method using humanoid robots did not have any significant disadvantages in terms of training success and acceptance compared to video and can offer advantages in certain areas of application.

The results also indicate no direct correlation between older age or greater professional experience and correct diagnosis. Accurate diagnoses were evenly distributed between medical students and nurses, suggesting that professional background is not a decisive factor. Overall, the participants felt less confident about assessing the human actor in the video. This uncertainty could be attributed to various factors. Possible reasons may include a lack of emotional intelligence and empathy in the video. However, the difference between the 2 groups in terms of self-perception was very small. The so-called uncanny valley effect describes the phenomenon in which humanlike robots that look and behave almost but not quite human evoke a feeling of discomfort or aversion in people. A low to medium human likeness is associated with increased positive responses, whereas a shift to negative responses is expected for highly anthropomorphic robots [21]. This feeling can make people feel more uncertain about how to assess the robot.

To address the research questions, participants evaluated the realism of symptom presentation and their ability to identify symptoms. Humanoid robots such as Ameca offer realistic movements and facial expressions through advanced sensors and actuators but remain limited to preprogrammed responses and lack spontaneity. While human actors present symptoms with greater emotional nuance, the robot showed comparable suitability due to its humanlike appearance and complex behavior. However, fully replicating human adaptability and expression remains technically challenging. In the future, large language models could generate patient-specific responses and replace video-based templates.

### Comparisons to Existing Literature

The results of our study show that the use of humanoid robots in training to simulate medical scenarios offers learners a more practical and low-risk training opportunity and is met with a high level of acceptance.

Comparable studies on the use of humanoid robots in medical scenarios or in training show similarly positive results in terms of learning success and acceptance among learners [22-24]. In a study by [23], the acceptance, effectiveness, and user-friendliness of an android robot patient was compared with that of a human actor patient for diagnostic teaching, training, and assessment. For this purpose, the android robot patient was implemented with features for simulating delirium. The results showed that an android robot patient can be used to provide safe training in assessing delirium diagnosis to participants with no previous knowledge. The android robot patient was not difficult for participants to use and led to greater confidence than using a human actor. Takanobu et al [24] developed a humanoid robot for training in dental therapy that can influence treatment through altered facial expressions or active movements of the neck and hands. This enabled students to undergo dental training that was closer to actual practice and avoid risks. Hashimoto et al [25] developed a humanoid robot named Saya. Saya’s speech and behavior can be remotely controlled by an operator. A field trial was conducted at an elementary school, where Saya led a science lesson on the principle of levers and interacted with the students.

### Limitations

The sample size of 21 participants was small, which limits the generalizability of the results. Furthermore, this was a pilot study with a specific study design that only allowed for a comparison between a specific humanoid robot and a video simulation. Other factors influencing the participants, such as previous technical experience, individual approaches to robots, or differences in clinical experience, were not systematically measured. The robot’s limited expressiveness due to the limitation of its facial muscles (number of motors in the face) may also have influenced the perception of the patient simulation. The assessment of acceptance and realism was based on participants’ individual subjective opinions, which are sensitive to bias.

### Implications

The results of this study show that humanoid robots achieved comparable acceptance and diagnostic accuracy to those of videos featuring actors playing patients. Humanoid robots offer a platform for individualized, artificial intelligence–supported learning processes and the simulation of rare or complex clinical pictures. At the same time, the results highlight the need for further technical development in terms of movement flexibility, facial expressions, and interaction capabilities. Future studies should include larger samples and different clinical pictures to comprehensively test effectiveness and acceptance.

### Conclusions

The results of our small pilot study show that the robot was not rated significantly worse by the participants than the video with the actor patient for training in the conduct of psychopathological assessments. In addition to the further technical development of the robot, future development should also take into account corresponding didactic theories and the design of the scenarios in which they are used. Furthermore, the training scenario can be transferred to various areas of application and institutions using the taxonomy by Bloom [20].

The humanoid robot patient embodies didactic concepts, and working with it could increase the motivation and commitment of learners and, at the same time, contribute to the development of problem-solving and teamwork skills. Compared to instructional videos, the robot offers the advantage that learning occurs constructively through working on real problems and knowledge is produced through interaction between people and with the environment.

## Data Availability

The datasets generated or analyzed during this study are available from the corresponding author on reasonable request.

## Appendix

Multimedia Appendix 1 Analysis of requirements.

Multimedia Appendix 2 Full questionnaire.

Multimedia Appendix 3 Psychopathological categories and associated symptoms assessed in the study and calculation for feasibility and usability study.
