# Supplementary material for: Comparing Patient Simulation With a Humanoid Robot or a Human Actor in Terms of Training Success and Acceptance: Pilot Questionnaire Study
Source: JMIR Form Res. 2025 Dec 5;9:e70363. doi: 10.2196/70363 (PMC12717505; doi:10.2196/70363)
Supplement: Multimedia Appendix 1 [file formative_v9i1e70363_app1.pdf]

Multimedia Appendix 1

Analysis of requirements

As part of the requirements analysis for a humanoid robot patient in medical education, the head of the simulated patient program at the University of Oldenburg was interviewed. The qualitative interview served to capture key assessments and experiences regarding the significance of simulated doctor-patient conversations.
He emphasized the high relevance of simulated doctor-patient conversations, as students can gain practical experience and make mistakes in a safe environment. Human simulated patients are superior in terms of emotional authenticity, while robots act more objectively and consistently. From an organizational standpoint, it is often difficult to provide the right actors for the right cases in a timely manner, especially with large numbers of students and a tight curriculum. The robot can serve as an additional training opportunity for symptom perception and is superior to textbook cases, especially for fact-based anamnesis or standardized tests. Limitations include limited mouth movements and a lack of facial expression subtlety. Tasks should be clearly structured and realistic, for example by having the robot in a seated position. The robot is particularly suitable for self-study and knowledge consolidation, has a high motivational value, and promotes equal opportunities, but offers less emotional depth than human actors. There is room for improvement in speech and lip movements, finer facial expressions, and slight facial asymmetry for greater naturalness.

The interview began with a general question about the relevance of patient simulation in medical education and current challenges in this area (I). Furthermore, a subjective assessment of the relevance of patient simulation by humanoid robots for recognizing symptoms was asked (II). There was a question on the assessment of the measures that should be applied with regard to the use of the simulated patient in education in order to achieve optimal comprehensibility of the task (clarity, complexity and visual representation and task selection). In the third block, he was asked what his personal assessment of the robotic simulation patient is with regard to the necessary practical relevance and integration into teaching (III). In block (IV), we asked for general recommendations for improving the robot for use as a simulation patient. In the last question (V), we ask about recommendations.

Relevance

Opening questions on the special aspects of simulating doctor-patient conversations in medical teaching and current challenges. We have summarized the answers to the first question regarding relevance below. Summary of the responses to the first question regarding relevance:
The simulation of doctor-patient conversations in medical education is very important, as students can gain a lot of experience and also make mistakes in a safe environment. It is important that the simulated patients (human actors) appear authentic and, in particular, emotionally portray the patient's background (e.g. life situation, stress). Challenges with simulated patients in the context of medical education are often to have the right patients available for the right cases at the desired times. This is often a challenge in terms of organization, as the curriculum is very tightly planned. There are also too many students, making it difficult to provide and maintain an appropriately high level of communication during the course. One advantage of the robot as a simulation patient is that it is more objective in examination situations than a human actor. The advantage of human actors is that they are more emotional and can, for example, show a

very specific mood that can be felt as soon as they enter the room (e.g. aggressive behavior, sadness, tension). Students should also learn to deal sensitively with emotional situations, e.g. to correctly interpret breaks in conversations when the patient is speaking.

Assessment

The answers to the first question regarding the assessment of the relevance of patient simulation by humanoid robots for recognizing symptoms are summarized in the following:

The emotional component is particularly important for psychiatry. Students should learn to establish a relationship of trust here, which is certainly more difficult with the robot. In purely fact-based terms, the robot has an advantage. This would be good as an additional exercise option, e.g. for training the identification of symptoms or clinical pictures. In contrast to case studies from textbooks, the robot would be more suitable. It was also noticed that the robot can only simulate mouth movements to a very limited extent, for example lip pursing is not possible. Fine movements of the lips are missing, as is flexibility in the jaw.

Tasks design

The answers to the question about the evaluation of measures to achieve optimal comprehensibility of the task (clarity, complexity and visual presentation, task selection) are summarized below:

The task design must be operationalizable. It should be possible to ask the robot about objectifiable factors, e.g. suicidality. Fact-based anamnesis interviews are well suited, also for other clinical disciplines. However, the robot should be able to sit in order to present the scenario more realistically. Standardized tests such as dementia tests are also suitable for practicing with the robot. In his opinion, however, an app for learning would be equally suitable.

Usability in practice

The head of the acting patient program was asked what he thinks about the practical relevance and integration of the robot patient into teaching. (Personal assessment of the practical relevance and integration into teaching).

The robot is well suited for self-study and for practicing theoretical knowledge. As an exercise method, the robot patient has a highly stimulating character, which is positive for students' motivation and learning success. However, the emotional component is reduced slightly by the robot. Equal opportunities among students are greater when practicing with the robot patient, as the robot patient is presented in the same way every time.

Recommendation

Finally, a recommendation was asked for:

Speech and lip movements should be optimized. The facial expressions could be made even finer. It would also be important for the two halves of the robot's face to be asymmetrical, as a symmetrical face would appear unnatural to humans. The robot should also be touchable. Since touching or holding the robot during operation can be dangerous, certain areas could perhaps be indicated with a laser. The robot should also be able to display pain reactions and vital signs (for other topics).
