# Supplementary material for: Comparing Patient Simulation With a Humanoid Robot or a Human Actor in Terms of Training Success and Acceptance: Pilot Questionnaire Study
Source: JMIR Form Res. 2025 Dec 5;9:e70363. doi: 10.2196/70363 (PMC12717505; doi:10.2196/70363)
Supplement: Multimedia Appendix 2 [file formative_v9i1e70363_app2.pdf]

Multimedia Appendix 2

Questionnaire

Table 2 shows the participant questionnaire used in the study to collect demographic data, professional background, and evaluation of the patient simulation. Items include age, gender, study program, work experience, and simulation type (robot or video). Participants rate the realism of symptoms, indicate whether all symptoms were identified, list any missed symptoms, report the number of symptoms observed before diagnosis, assess confidence in their diagnosis, and specify the diagnosis made.

Age: __________________ **Gender**: male O female O

Study program: Medicine O Psychology O

Work experience available: O yes,____ years no O

Patient simulation by: **Robot** O **Video** O

	For each statement, please indicate how much you agree with it.	1 = Fully agree<br>2 = Agree somewhat<br>3 = Strongly disagree<br>4 = Do not agree<br>5 = Do not agree at all				
		1	2	3	4	5
1.	How realistically were the symptoms presented?	Very realistic ---> unrealistic<br>O O O O O				
2.	Were you able to identify all the symptoms presented?	yes O no O				
2.a.	Which symptoms could not be identified?	Nr.:_____				
3.	How many symptoms were presented to you by the simulation patient before you made a diagnosis?	Number:____				
4.	How sure are you that the diagnosis you have made is correct?	Very safe -----> unsafe<br>O O O O O				
5.	What diagnosis have you made? ________________________________					
