# Supplementary material for: Comparing Patient Simulation With a Humanoid Robot or a Human Actor in Terms of Training Success and Acceptance: Pilot Questionnaire Study
Source: JMIR Form Res. 2025 Dec 5;9:e70363. doi: 10.2196/70363 (PMC12717505; doi:10.2196/70363)
Supplement: Multimedia Appendix 3 [file formative_v9i1e70363_app3.pdf]

Multimedia Appendix 3

Psychopathological categories and associated symptoms assessed in the study and Calculation for feasibility and usability study.

Psychopathological findings

Table 1 lists the psychopathological categories and associated symptoms assessed in the study. The domains include attention and memory, formal thinking, compulsions and fears, delusion and substantive thinking, sensory illusions, ego disorders, and affectivity. Each domain contains specific symptom descriptors used for evaluation during the patient simulations.

Table 1: Psychopathological categories and associated symptoms assessed in the study

**Attention and memory**<br>Concentration / memory and memory disorders / comprehension disorders / concentration disorder
**Formal thinking**<br>Slowed/ rambling/inhibited/ cumbersome/ restricted/ perseveration/ brooding/ insistent thoughts/ flight of ideas/ talking past the point/ blocked/ breaking off thoughts/ incoherent, disjointed/ neologisms
**Compulsions and fears**<br>Obsessive thoughts/ compulsive actions/ compulsive impulses/ phobias/ panic attacks/ fears
**Delusion and substantive thinking**<br>Delusion/ delusional idea/ delusional thought/ delusional perception/ delusional mood/ delusional guilt/ delusional impoverishment/ persecution mania/ impairment delusional relationship delusion/ hypochondriacal delusion/ overvalued ideas
**Sensory illusions**<br>Illusions/acoustic, optical, olfactory, gustatory, tactile, haptic Hallucinations/ zoenesthesia
**Ego disorders**<br>Derealization/ depersonalization/ thought expansion/ withdrawal/ inspiration/ physical and other experiences of external influence
**Affectivity**<br>Mood noticeably changed/ helplessness/ feeling of numbness/ low affect/ depressed, depressive/ hopeless/ anxious/ euphoric/ dysphoric/ irritable/ complaining/ feelings of inadequacy/ increased self-esteem/ feelings of guilt/ ambivalent/ parathymia/ affectively labile/ affectively incontinent/ affectively rigid/ ability to oscillate well/ reduced not preserved

User acceptance study

Table 2 compares mean scores from participant evaluations of the established simulation method (video with human actor) and the new method (humanoid robot). The table reports average ratings for perceived realism of symptoms, ability to identify all symptoms, number of symptoms observed before diagnosis, confidence in the diagnosis, and diagnostic accuracy. Overall mean values and the lower tolerable deviation threshold are also provided.

Table 2: User acceptance study

No. Question		Established procedure: Video (Mean value)	New procedure: Robot (Mean value)
1	How realistic were the symptoms presented?	4.8	4
2	Have you been able to identify all the symptoms shown?	0.9	0.9
3	How many symptoms were presented to you by the simulation patient before you made a diagnosis?	4.2	3.5
4	How sure are you that the diagnosis you have made is correct?	4.1	4.2
5	Diagnosis correct?	0.9	0.9
	Total mean value	2.98	2.7
	Lower tolerable deviation (20%)	-0.6	

New procedure: Robot					
Question:	**1**	**2**	**3**	**4**	**5**
	3	0	5	1	1
	3	1	3	5	1
	4	1	5	5	1
	4	1	5	4	1
	4	1	5	2	0
	3	1	3	5	1
	5	1	3	5	1
	5	1	3	5	1
	5	1	2	5	1
	4	1	2	5	1
	4	1	3	5	1
mean value:	4	0.9	3.5	4.2	0.9
Established procedure: Video					
Question:	**1**	**2**	**3**	**4**	**5**

	4	1	2	5	1
	5	1	5	5	1
	5	1	5	4	1
	5	1	5	3	1
	5	1	2	5	1
	5	1	5	4	1
	5	1	5	4	1
	5	1	5	2	0
	4	1	3	4	1
	5	0	5	5	1
mean value:	4	0.9	4.2	4.1	0.9
